# Uncovering Dynamics of End-of-Life Decision Making Among Individuals at Risk of ADRD: Insights from Clinical Data

**DOI:** 10.1093/geroni/igaf122.4251

**Published:** 2025-12-31

**Authors:** Shaoqing Ge, Qingyi Jasmine Zeng, Katherine Britt, Bin Huang

**Affiliations:** University of Texas at Austin School of Nursing, Austin, Texas, United States; The University of Texas School of Nursing, Austin, Texas, United States; University of Iowa, Iowa City, Iowa, United States; BrainCheck Inc, Austin, Texas, United States

## Abstract

This study examined how personal and relational factors from both patients and their caregivers influence advance care planning (ACP) decisions in real-world clinical settings. We focused on four outcomes central to end-of-life decision-making: discussion of end-of-life wishes, designation of financial and health care powers of attorney, and recognition of palliative or hospice care appropriateness. We analyzed data from individuals aged 40 and older who completed computerized cognitive care planning following cognitive assessment failures (n = 522). We applied logistic regression models to identify predictors of ACP engagement, incorporating variables from both patients and caregivers. Among patient-related factors, older age and higher education were consistently associated with increased ACP engagement. In contrast, patients identifying as Non-Hispanic Black or Hispanic/Latino were significantly less likely to engage in end-of-life discussions or designate proxies. Lower functional status also predicted greater planning activity. Caregiver-related predictors revealed that moderate stress and awareness of support resources were positively associated with proxy designation and hospice recognition. However, frequent weekly contact with the patient and perceived ability to provide care were negatively associated with ACP outcomes. Higher caregiver depression scores were linked to a reduced likelihood of health care proxy designation. These findings highlight the importance of dyadic and contextual influences in shaping ACP decisions. Emotional readiness, relational dynamics, and caregiving burden play critical roles in whether and how planning occurs. Our results underscore the need for interventions that recognize the shared nature of end-of-life decision-making and support both parties in navigating the emotional and interpersonal challenges of caregiving.

